# Comparing Perinatal Outcomes of Assisted Reproductive Technology (ART)-Induced vs. Naturally Conceived Twin Pregnancies

**DOI:** 10.7759/cureus.69842

**Published:** 2024-09-21

**Authors:** Paraskevas Perros, Antonios Koutras, Ioannis Prokopakis, Zacharias Fasoulakis, Thomas Ntounis, Gerasimos Boulieris, Eirini Geramani, Vasilios Lygizos, Maria Fanaki, Angelis Peteinaris, Vasilios Pergialiotis, Panagiotis Antsaklis, Konstantinos Daglas, Athanasios Chionis, Emmanuel Kontomanolis, George Daskalakis

**Affiliations:** 1 Department of Obstetrics and Gynecology, General Hospital of Athens ‘Alexandra Hospital’, National and Kapodistrian University of Athens, Athens, GRC; 2 Department of Urology, University of Patras, Patras, GRC; 3 Department of Obstetrics and Gynecology, Laiko General Hospital of Athens, Athens, GRC; 4 Department of Obstetrics and Gynecology, Democritus University, Alexandroupolis, GRC

**Keywords:** assisted reproductive technology (art), cesarean section (cs), low birth weight (lbw), mortality, multiple pregnancy, preterm birth (pb)

## Abstract

The occurrence of multiple pregnancies is consistently and significantly linked to the growing use of assisted reproductive technology (ART). Even very young women opt for having multiple embryos implanted by medical professionals in order to increase the chances of a successful outcome. Our aim is to review the research on cesarean section rates and perinatal outcomes, like perinatal morbidity, risk of preterm delivery, and low birth weight (LBW) of neonates in multiple pregnancies that resulted from ART in comparison to those that were naturally conceived. We conducted a comprehensive search of the PubMed, Crossref, and Google Scholar electronic databases for related articles up to January 2024 using the Preferred Reporting Items for Systematic Reviews and Meta-Analyses (PRISMA) guidelines. We found that most studies found no relationship between ART procedures and poor perinatal outcomes in multiple pregnancies compared to naturally conceived ones. A few studies have linked ART services to preterm birth (PTB) and LBW. Careful interpretation of these findings is necessary since confounding factors may invalidate the putative link. Although perinatal death rates are similar, ART increases cesarean section rates. When a statistically significant difference was detected, it was typically attributable to confounding variables such as maternal age, subfertility reasons, or maternal comorbidities like gestational diabetes or hypertension.

## Introduction and background

The proportion of children conceived through the use of assisted reproductive technology (ART) consistently and significantly rises alongside the incidence of multiple pregnancies [1, 2]. Clinicians frequently transfer multiple embryos in an effort to increase the probability of success, given the typically high cost of in vitro fertilization (IVF) treatments [3], a trend that even relatively young women elect to participate in. Multiple pregnancies occur at a greater rate in ART pregnancies than in naturally conceived ones [4].

It is commonly acknowledged that twin pregnancies, regardless of whether they are monochorionic or dichorionic, are linked to a greater risk of maternal complications compared to singleton pregnancies [5]. The underlying diagnosis of infertility may have an effect on neonatal outcomes as well as maternal and delivery characteristics; however, the relative obstetrical risks of twin pregnancies that are conceived naturally and those that are conceived by IVF are not yet known.

A variety of studies have been conducted to investigate the differences in neonatal outcomes and mother characteristics between pregnancies that were conceived with ART and those that were conceived spontaneously. The majority of reports, on the other hand, have concentrated on singleton pregnancies [6-8]. Very few studies have particularly studied the maternal features and newborn outcomes in twin pregnancies that were the result of ART.

The purpose of this analysis is to evaluate perinatal outcomes, specifically the perinatal morbidity, risk of preterm delivery, and low birth weight (LBW) of neonates in multiple pregnancies that resulted from ART in comparison to those that were naturally conceived. The second factor that is investigated is the percentage of cesarean sections. In order to determine the relationship between birth outcomes and assisted conception, we searched at any published study that differentiated between singleton and multiple births and included a sufficient control group from a population that was comparable.

Materials and methods

We performed a comprehensive search in electronic databases Pubmed/Medline, Scopus, and Google Scholar until January 2024. The search encompassed a variety of topics, including perinatal morbidity, ART, IVF, intracytoplasmic sperm injection (ICSI), multiple pregnancy, dichorionic diamniotic (DCDA) pregnancies, preterm birth (PTB), and LBW.

Irrespective of the content availability and publication time, all articles containing an English title and abstract were admitted at the outset. As a result of the preliminary investigation, 258 publications were obtained. The abstracts and titles of the publications that were obtained were evaluated by two independent reviewers.

**Figure 1:**
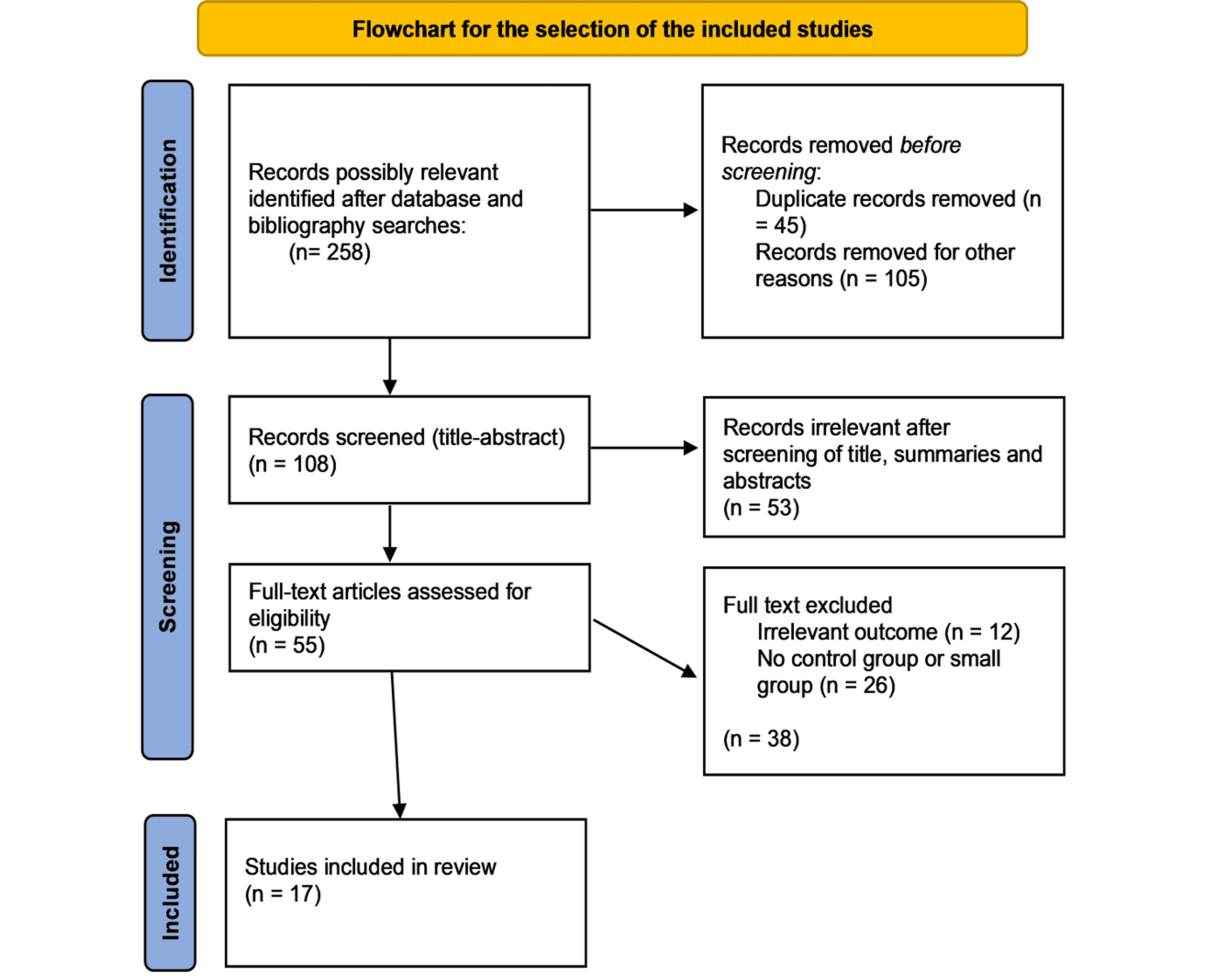
A PRISMA flowchart outlining the study selection process PRISMA: Preferred Reporting Items for Systematic Reviews and Meta-Analyses

A total of 55 full-text articles were obtained, and 17 studies were chosen for data extraction following a thorough evaluation. Only original publications, case reports, cohort studies, and case series met our inclusion criteria. Articles in animal models, systematic reviews, and meta-analyses, or published in a language other than English were excluded. The flow diagram illustrated in Figure 1 provides a schematic representation of the various phases involved in article selection. 

## Review

Results

Twin pregnancies, regardless of the method of conception, are associated with an elevated risk of morbidity and mortality for both the mother and the child. Consequently, couples should be advised of the elevated risk of iatrogenic twinning that is associated with double embryo transfer. We collected and presented recent studies to investigate the potential risk of PTB and LBW, perinatal morbidity, and the rates of cesarean section in twins conceived after using ART compared to the general population.

The results were conflicting, as there were many studies that did not show an impaired perinatal outcome in multiple pregnancies compared to the general population, but also studies that resulted in a difference. This is probably related to the heterogeneity of the populations studied as well as to the heterogeneity of the approach to twin pregnancies [9-12].

Risk of PTB and LBW

In Europe, PTB is characterized as delivery occurring between 22+0 and 37+0 weeks of gestation with a prevalence of 5% to 18%, when just 0.3%-0.5% of them happen prior to the 28^th^ week of gestation. Preterm neonates are categorized into three groups based on gestational age at delivery: 24+0-27+6 weeks (extremely preterm), 28+0-31+6 weeks (early preterm), and 32+0-36+6 weeks of gestation (moderate-to-late preterm) [13]. Low birth weight is considered a newborn with a weight under 2500 g and very LBW with a weight under 1500 g. A variety of studies assessed the risk of PTB and LBW in multiple pregnancies conceived using ART compared to those conceived naturally.

Dhont et al. [14] analyzed 357 singleton pregnancies and 1,241 multiple, born between 1992 and 1997, with 90% of them resulting from IVF. The authors concluded that among singleton pregnancies, perinatal outcomes (PTB, LBW) in the IVF group were worse, but when it was coming for multiple pregnancies, the results were comparable. Luke et al. [15] also compared the perinatal outcome in twin pregnancies resulting from ART and spontaneous conception. The study included cases from four tertiary hospitals with a total of 2,143 spontaneous conception and 424 assisted conception pregnancies. The final conclusions showed no statistically significant difference between the two groups in terms of prematurity and birth weight.

Furthermore, no significant statistical difference was identified in rates of PTB and LBW by Vasario et al. [16], who compared 223 twin pregnancies (84 after IVF and 139 from spontaneous conception), and by Ágústsson et al. [17], who compared 453 naturally conceived multiple pregnancies and 69 after ART. In accordance with them was also the study by Olivennes et al. [18], who conducted a retrospective study that consisted of children born either from IVF (n = 72), spontaneous conception after ovarian stimulation (n = 82), or spontaneous conception (n = 164). Similar results were also reported in a retrospective study held by Choi et al. [19] and Huang et al. [20] from Taipei University Hospital, who examined a total of 194 twin pregnancies (50 from spontaneous conceptions, 63 from intrauterine insemination (IUI), and 81 from IVF/ICSI). Another study by Zuppa et al. [21] reported comparable perinatal outcomes in twin pregnancies when analyzing data from 388 neonates (100 spontaneously conceived, 126 from IUI, and 162 by IVF/ICSI). Similar results were also reported by Bensdorp et al. [22]. Last but not least, a prospective observational study by Domingues et al. [13] reported no significant differences among induced and naturally conceived twin pregnancies. The findings indicated that preterm premature rupture of membranes occurred less frequently in the ART group (23.9% vs. 12.8%).

However, Bernasko et al. [23] analyzed the perinatal outcome in twin pregnancies, collecting 105 twin pregnancies after IVF or gamete intrafallopian transfer (GIFT) and 279 by natural conception, born in the period 1990-1995. reporting increased rates of low birth weight in the IVF/GIFT group but no statistically significant increase in terms of preterm delivery.

On the other hand, Arian et al. [12] reported slightly increased rates of PTB and LBW in the ART group when compared with spontaneously conceived twin pregnancies. The study was held in the United States and referred to births during the period 2015-2017, including 34,086 ART pregnancies and 320,638 naturally conceived ones. Morcel et al. [24] reported elevated rates of very preterm births (odds ratio (OR) 2.20, 95% CI 1.02-4.77, p<0.05), LBW (1.77, 1.21-2.61, p<0.01), very low birth weight (1.99, 1.13-3.49, p<0.05), and neonatal intensive care unit (NICU) admissions (1.66, 1.14-2.43, p<0.01) in their retrospective study of 350 twin DCDA pregnancies delivered at ≥22 weeks of gestation from 2001 to 2005 in a tertiary maternity unit. Ovarian induction was linked to an elevated risk of preterm and very preterm births (2.25, 1.06-4.75, p<0.05 and 3.47, 1.42-8.49, p<0.01, respectively), low and very low birth weights (2.87, 1.63-5.05, p<0.001 and 2.59, 1.33-5.07, p<0.01, respectively), NICU admission (2.92, 1.67-5.11, p<0.001), and fetal or neonatal mortality (4.20, 1.40-12.56, p<0.05). The mean gestational age (p<0.001) and mean birth weight of the first (p<0.01) and second twins (p<0.001) were diminished in the ovarian induction (OI) group. Furthermore, adverse perinatal outcomes were examined by Moini et al. [25] in their prospective cohort study in the ART group. The authors at Arash Women's Hospital analyzed every dichorionic twin pregnancy in nulliparous women after fresh IVF or ICSI cycles at Royan Institute (n = 320) with naturally conceived ones (n = 170), from January 2008 to October 2010 [25]. In accordance with them were the retrospective studies by Daniel et al. [26] and by Geisler et al. [27]. The final cohort comprised all viable DCDA twin pregnancies (n = 539) born in Cork University Maternity Hospital, Ireland, from 2009 to 2012. Of these, 171 women (31.7%) conceived with ART, while 368 women (68.3%) conceived spontaneously. Within the ART group, 109 individuals (63.7%) achieved conception via IVF treatment, 20.5% (n = 35) with ICSI treatment, and 15.8% (n = 27) utilized donated oocytes for IVF/ICSI. Twins conceived with ART were approximately twice as likely to be delivered moderately preterm (32-33+6w) and have respiratory distress syndrome (RDS). No differences were observed in the incidence of LBW and very low birth weight infants based on the mode of conception [28].

In conclusion, perinatal outcomes of IVF twin pregnancies, when compared to spontaneously conceived twin pregnancies, are controversial. The majority of the studies [13-22] claimed comparable results among the two groups. Nevertheless, part of the literature [24-26] revealed an increased risk of PTB and LBW regarding ART pregnancies. It is important to point out that in the study by Morcel et al. [26], rates of PTB and LBW were increased in the OI group, while in the Moini et al. [27] prospective cohort study, the ART group used ICSI to achieve conception.

Furthermore, in the study conducted by Arian et al. [12], rates were slightly increased with no statistical significance. The results were conflicting, and this is probably related to the heterogeneity of the populations studied, as well as to the heterogeneity of the approach to twin pregnancies, the type of infertility, and the different ART procedures used.

All the aforementioned studies that analyzed the risk of PTB and LBW are summarised in Table 1.

**Table 1:** Studies analyzed in the review regarding risk of preterm birth (PTB) and low birth weight (LBW) DCDA: dichorionic diamniotic; ART: assisted reproductive technology; NC: naturally conceived

Authors	Type of study	Chorionicity	Period of birth	ART	NC	Risk of PTB	Risk of LBW
Dhont et al. [14]	Case-control	All	1992-1997	1100	141	No difference	No difference
Luke et al. [15]	Observational	All	1990-2002	424	2143	No difference	No difference
Vasario et al. [16]	Prospective	DCDA	2004-2008	84	139	No difference	No difference
Ágústsson et al. [17]	Retrospective	All	1990-1993	69	453	No difference	No difference
Olivennes et al. [18]	Retrospective	Αll	1988-1993	154	164	No difference	No difference
Choi et al. [19]	Retrospective	All	1994-2003	206	392	No difference	No difference
Huang et al. [20]	Retrospective	All	1992-2001	144	50	No difference	No difference
Zuppa et al. [21]	Retrospective	All	1988-1997	288	100	No difference	No difference
Bensdorp et al. [22]	Prospective	DCDA	2000-2012	3418	3276	No difference	No difference
Domingues et al. [13]	Prospective	All	1996-2011	180	698	No difference	No difference
Bernasko et al. [23]	Retrospective	All	1990-1995	105	279	No difference	Increased in the ART group
Arian et al. [12]	Retrospective	All	2015-2017	34086	320638	Slightly increased in the ART group	Slightly increased in the ART group
Morcel et al. [24]	Retrospective	DCDA	2001-2005	104	246	Increased in the ART group	Increased in the ART group
Moini et al. [25]	Prospective	DCDA	2008-2010	320	170	Increased in the ART group	Increased in the ART group
Daniel et al. [26]	Retrospective	All	1996-1997	104	193	Increased in the ART group	Increased in the ART group
Geisler et al. [27]	Retrospective	DCDA	2009-2012	171	368	Increased in the ART group	No difference

Perinatal Mortality Rates

Twin pregnancies typically present a heightened risk of morbidity and mortality for both the mother and infants in comparison to singleton pregnancies; consequently, couples should receive appropriate counseling [5]. Concerning the increased likelihood of iatrogenic twinning due to double embryo transfer, it is crucial to assess whether the already elevated risk of multiple pregnancies is exacerbated by assisted conception techniques [3].

A retrospective study by Ágústsson et al. [17] reported comparable results as far as perinatal morbidity rates are concerned. Similar results were also stated in other retrospective studies, conducted by Bernasko et al. [23], Olivennes et al. [18], and Choi et al. [19], who studied and compared perinatal mortality in twin pregnancies conceived using ART and those after natural conception. Comparable perinatal mortality rates were also found by Dhont et al. [14] in their comparative study and by Huang et al. [20], from Taipei University Hospital, who examined 194 twin pregnancies, born during the period 1992-2001, excluding those with preeclampsia or gestational diabetes mellitus. In accordance with the previous conclusions were the retrospective studies by Zuppa et al. [21], who analyzed data from 388 neonates (100 by spontaneous conception, 126 by IUI, and 162 by IVF/ICSI), and Daniel et al. [26], who collected all live twin pregnancies (delivery ≥24 weeks) between 1996 and 1997 in Israel. Furthermore, no statistical difference in perinatal morbidity in twin pregnancies after ART and natural conception was reported in a prospective study held by Domingues et al. [13] and another retrospective study by Bensdorp et al. [22]. Finally, Geisler et al. [27] confirmed that the mode of conception had no impact on the rate of perinatal death in multiple pregnancies.

On the other hand, Lambalk et al. [28] conducted a retrospective study that analyzed data from the German national healthcare system, comparing perinatal outcomes of twin pregnancies of spontaneous conception with ART pregnancies. They concluded that despite all the other factors, the ART group was at an increased risk of perinatal morbidity. In accordance with them were also the different retrospective studies held by Morcel et al. [24] and Moini et al. [25], who compared all dichorionic twin pregnancies in nulliparous women. Additionally, Arian et al. [12], in a population-based investigation of twin pregnancies in the USA, identified an elevated risk of newborn morbidity among twins conceived using reproductive procedures compared to those conceived spontaneously.

All the aforementioned studies that analyzed perinatal mortality rates are summarized in Table 2.

**Table 2:** Studies on perinatal mortality rates analyzed in the review DCDA: dichorionic diamniotic; ART: assisted reproductive technology; NC: naturally conceived

Authors	Type of study	Chorionicity	Period of birth	ART	NC	Perinatal mortality
Ágústsson et al. [17]	Retrospective	All	1990-1993	69	453	No difference
Bernasko et al. [24]	Retrospective	All	1990-1995	105	279	No difference
Olivennes et al. [18]	Retrospective	Αll	1988-1993	154	164	No difference
Choi et al. [19]	Retrospective	All	1994-2003	206	392	No difference
Dhont et al. [14]	Case control	All	1992-1997	1100	141	No difference
Huang et al. [20]	Retrospective	All	1992-2001	144	50	No difference
Zuppa et al. [21]	Retrospective	All	1988-1997	288	100	No difference
Luke et al. [15]	Retrospective	All	1990-2002	424	2143	No difference
Daniel et al. [27]	Retrospective	All	1996-1997	104	193	No difference
Domingues et al. [13]	Prospective	All	1996-2011	180	698	No difference
Geisler et al. [27]	Retrospective	DCDA	2009-2012	171	368	No difference
Lambalk et al. [28]	Retrospective	All	1994-1996	480	613	Increased in the ART group
Morcel et al. [24]	Retrospective	DCDA	2001-2005	104	246	Increased in the ART group
Moini et al. [25]	Prospective	DCDA	2008-2010	320	170	Increased in the ART group
Arian et al. [12]	Retrospective	All	2015-2017	34086	320638	Increased in the ART group

Rates of Cesarean Section

Another important factor that was examined was the rate of cesarean section and if it can be altered in twin pregnancies according to the mode of conception. 

Most of the previously analyzed studies also investigated the cesarean section rates. Domingues et al. [13], in their prospective observational study, observed a higher cesarean section rate (50.6% vs. 63.9%). The comparison involved 180 induced twins and 698 spontaneously conceived. Furthermore, Bernasko et al. [23], in their study of 105 twin pregnancies after IVF or GIFT and 279 by natural conception, also reported increased rates of elective cesarean sections in the IVF/GIFT group. In accordance with them were Olivennes et al. [18] in their retrospective study, as they observed a non-statistically significant difference between the two groups, the only exception being emergency cesarean section rates, which were elevated in the IVF group. Moreover, Geisler et al. [28], in their retrospective study of all viable DCDA twin pregnancies (n = 539) delivered at Cork University Maternity Hospital, found the ART twins’ group exhibited a twofold increased likelihood of being delivered via cesarean section (OR 2.35; 95% CI 1.76-3.14). Increased rates of cesarean section in the ART twin pregnancies were also stated in retrospective studies conducted by Daniel et al. [26] and Vasario et al. [16]. Monochorionic and triplet pregnancies were not included. Similar findings were reported in a comparable study by Ágústsson et al. [17], held between 1990 and 1993, that consisted of 453 naturally conceived multiple pregnancies and 69 after ART.

On the other hand, we tracked three studies that claimed no increased cesarean section rates. Firstly, the retrospective study by Lambalk et al. [28], included data from 613 naturally conceived twin pregnancies and 480 from IVF. Secondly, the comparative study by Dhont et al. [14], held in the German sector of Belgium from 1992-1997, studied 1,241 twins, 90% of them being the result of IVF, as well as Bensdorp et al. [22], who compared dizygotic twin pregnancies: 6,694 women, 470 after OI, 511 after intrauterine insemination with controlled ovarian hyperstimulation (IUI-COH), 2,437 after IVF with ICSI, and 3,276 after natural conception, and concluded to no differences in cesarean section rates.

All the aforementioned studies regarding cesarean section rates are summarized in Table 3.

**Table 3:** Studies included in the review regarding cesarean section rates DCDA: dichorionic diamniotic; ART: assisted reproductive technology; NC: naturally conceived

Authors	Type of study	Chorionicity	Period of birth	ART	NC	Rates of cesarean section
Domingues et al. [13]	Prospective	All	1996-2011	180	698	Increased in the ART group
Bernasko et al. [23]	Retrospective	All	1990-1995	105	279	Increased in the ART group
Olivennes et al. [18]	Retrospective	All		154	164	Increased in the ART group
Geisler et al. [27]	Retrospective	DCDA	2009-2012	171	368	Increased in the ART group
Daniel et al. [26]	Retrospective	All	1996-1997	104	193	Increased in the ART group
Ágústsson et al. [17]	Retrospective	All	1990-1993	69	453	Increased in the ART group
Vasario et al. [16]	Prospective	DCDA	2004-2008	84	139	Slightly Increased in the ART group
Lambalk et al. [28]	Retrospective	All	1994-1996	480	613	No difference
Dhont et al. [14]	Case-control	All	1992-1997	1100	141	No difference
Bensdorp et al. [22]	Prospective	DCDA	2000-2012	3418	3276	No difference

Discussion

The utilization of ART has resulted in a rise in the occurrence of multiple pregnancies. A twin pregnancy is a high-risk state that may lead to health complications both for the mother (i.e., pregnancy-induced hypertension, anemia, blood clotting issues, sepsis from premature rupture of membranes, and postpartum hemorrhage) and for the babies (i.e., premature birth, premature rupture of membranes, and fetal growth restriction) compared to a single pregnancy [5, 29, 30]. The practice of transferring several embryos, either two or three, is being increasingly supplanted by the strategy of transferring a single embryo, with the aim of minimizing the occurrence of multiple pregnancies and the associated negative consequences [31].

As a result, it is important to investigate how the use of ART might be affecting the perinatal and neonatal outcomes in multiple pregnancies.

The purpose of this review is to analyze the perinatal outcomes, specifically the perinatal morbidity, risk of preterm delivery, and LBW of neonates in multiple pregnancies that resulted from ART in comparison to those that were naturally conceived. The second aspect that is examined is the percentage of cesarean sections. To determine the relationship between birth outcomes and assisted conception, we looked at any published study that differentiated between singleton and multiple births and included a sufficient control group from a population that was comparable.

Differences in perinatal outcomes, such as PTB and LBW, of ART twin pregnancies when compared to spontaneously conceived twin pregnancies were controversial. Most of the studies [13-23] reported comparable results among the two groups. Nevertheless, part of the literature [24-26] revealed increased risk of PTB and LBW in ART pregnancies. It is important to point out that in the Morcel et al. [24] study, rates of PTB and LBW were increased in the OI group, and in the Moini et al. [25], prospective cohort study, the ART group used the ICSI method to achieve conception.

Furthermore, in the Arian et al. [12] study, rates were slightly increased with no statistical significance, and we tracked also two studies, one by Bernasko et al. [23], who reported only increased risk of LBW, and the other by Geisler et al. [27], who stated only elevated rates of PTB. The results were conflicting; this is probably related to the heterogeneity of the populations studied as well as to the heterogeneity of the approach to twin pregnancies, the type of infertility, and the different ART procedures.

The risk of unfavorable composite neonatal outcomes was marginally reduced in the spontaneous conception live twin birth cohort, even after accounting for many possible cofactors. Considering the low absolute risks of maternal and neonatal morbidity, overall reassurance regarding these outcomes can be provided to the patients undergoing all types of fertility treatments.

Perinatal mortality is another factor that concerns parents when it comes to multiple pregnancies. Reviewing the latest data, we observed that most of the studies [13, 14, 15, 17, 21, 23, 26, 27] did not reveal any elevated risk of perinatal mortality among twin pregnancies when the mode of conception is examined. Elevated risk was only reported by four studies [12, 24, 25, 28].

Cesarean delivery is one of the most common surgical procedures performed in the United States [32]. Although very commonly performed, it is not without risk when compared with vaginal delivery. Consequently, the mode of delivery poses an important concern, as well as if and how its rate is related to the mode of conception. Cesarean section rates were increased in the ART group in most of the studies [13, 16-18, 23, 26, 27], compared with the general population and spontaneously conceived multiple pregnancies, when only three reported comparable results [13, 14, 22-28]. The largest percentage of cesarean sections in the induction group, as described by other studies [13], could be attributed to a greater parental anxiety and obstetric stress surrounding these pregnancies. Furthermore, maternal age is also increased in the ART group, and so are, consequently, the maternal comorbidities, like gestational diabetes or hypertension.

## Conclusions

The majority of the literature shows no association between ART procedures and adverse perinatal outcomes in multiple pregnancies when compared with naturally conceived ones. However, there are a small number of studies that have demonstrated possible associations between various ART procedures and the risk of PTB and LBW. It is fundamental that careful consideration be given to the interpretation of these findings, as taking into account any confounding factors may render the purported association invalid. Similarly, the outcomes as far as perinatal mortality rates are concerned are similar with the general population, while on the other hand, cesarean section rates are elevated in the ART group.
